# NSAID Treatment Before and on the Early Onset of Acute Kidney Injury Had an Opposite Effect on the Outcome of Patients With AKI

**DOI:** 10.3389/fphar.2022.843210

**Published:** 2022-05-17

**Authors:** Hai Wang, Tong Liu, Qinglin Li, Ruixia Cui, Xueying Fan, Yingmu Tong, Shuzhen Ma, Chang Liu, Jingyao Zhang

**Affiliations:** ^1^ Department of Hepatobiliary Surgery, The First Affiliated Hospital of Xi’an Jiaotong University, Shaanxi, China; ^2^ Department of Laboratory Medicine Center, Xi’an People’s Hospital (The Fourth Hospital of Xi’an), Shaanxi, China; ^3^ Health Management Department, The Second Affiliated Hospital of Xi’an Jiaotong University, Shaanxi, China; ^4^ Department of Surgical ICU (SICU), The First Affiliated Hospital of Xi’an Jiaotong University, Shaanxi, China

**Keywords:** non-steroidal anti-inflammatory drugs, acute kidney injury, AKI progression, dialysis, discharge to home, 14-day mortality

## Abstract

**Background:** NSAIDs are one of the most frequently used medications and a risk factor for AKI. However, the optimal time of NSAIDs in patients with AKI is unknown.

**Methods:** A secondary analysis of a multicenter, randomized clinical trial including adult inpatients with acute kidney injury was performed. Univariate, multivariate, and subgroup analyses were used to explore the impact of NSAIDs during the early onset of AKI on the outcome of patients with AKI.

**Results:** A total of 6,030 patients with AKI were enrolled in the study. Following are the findings of the multi-factor analysis: NSAID treatments within 72 and 24 h before the onset of AKI were not associated with AKI progression, dialysis, or discharge from dialysis; only NSAID treatment within the 24-h onset of AKI was associated with these outcomes, and their OR values were independently 1.50 (95% CI: 1.02–2.19, *p* = 0.037), 4.20 (95% CI: 1.47–11.97, *p* = 0.007), and 0.71 (95% CI: 0.54–0.92, *p* = 0.011); only NSAID treatment within the 24-h onset of AKI would decrease the 14-day mortality, and the OR value was 0.52 (95% CI: 0.33–0.82, *p* = 0.005). The subgroup analysis revealed that in patients with age ≥65 years, CKD (chronic kidney disease), congestive heart failure, hypertension, and liver disease, NSAID treatments within the 24-h onset of AKI would deteriorate the outcome of patients with AKI.

**Conclusion:** Before an early onset of AKI, NSAID treatment might be safe, but during the onset of AKI, even early NSAID treatment would deteriorate the outcome of patients with AKI.

## Background

NSAIDs are one of the most often prescribed medications globally, accounting for around 5% of all prescribed medications (Bindu et al., 2020). NSAIDs are frequently used to treat rheumatic illnesses, rheumatoid arthritis, various acute and chronic pain, perioperative analgesia, and acute and chronic fever (Ghlichloo and Gerriets, 2021). Furthermore, the role of low-dose aspirin in the secondary prevention of ischemic stroke is well established (Frontera et al., 2016; Lip et al., 2018; Jia et al., 2021), and several studies have demonstrated that NSAIDs may be beneficial in preventing several cancers, including colorectal cancer, ovarian cancer, breast cancer, and prostate cancer (Ye et al., 2020; Kolawole and Kashfi, 2022).

The estimates of AKI prevalence range from <1% to 66% (Hoste et al., 2018), whereas AKI occurs at a rate of 10% in emergency patients, and its hospital mortality is also 10% (Challiner et al., 2014). The overall incidence of AKI is higher in ICUs (intensive care units), ranging from 20% to 50%, and the associated mortality is more than 50% (Selby et al., 2012). NSAIDs can result in renal damage, including AKI and CKD, depending on the dosage and duration of usage, particularly in older individuals and patients with comorbidities (Baker and Perazella, 2020). Due to the broad effects of NSAIDs, they are one of the most often prescribed medications (Bindu et al., 2020).

To summarize, NSAIDs increase the risk of AKI and CKD, and there is consensus on their use in CKD. However, there is a paucity of information about NSAID effects on the AKI progression and the prognosis of patients with AKI at different periods of AKI. Consequently, additional investigation into the impact of NSAIDs on AKI development and prognosis in individuals with AKI is required.

### Objective

The study aimed to explore the effects of NSAIDs before and after an early onset of AKI on AKI progression and the prognosis of patients with AKI.

## Methods

### Study Design

A secondary analysis of a multicenter randomized clinical trial including adult inpatients with acute kidney injury was performed (Trial registration: ClinicalTrials.gov NCT02753751).

### Setting

Six hospitals in the Yale New Haven Health System in Connecticut and Rhode Island, United States, were selected, including Bridgeport Hospital, Greenwich Hospital, The Hospital of St. Raphael, Lawrence and Memorial Hospital, Yale New Haven Hospital, and Westerly Hospital.

### Ethics Approval and Consent to Participate

The original author had obtained the ethical approval prior to conducting this research, and our study involved retrospective data analysis. A new ethical approval was approved by the Medical Bioethics Committee, School of Medicine, Xi’an Jiaotong University, Xi’an, Shaanxi province, China (No. 2022-468).

### Data Source

The data used in our study were shared by Wilson, Francis, Yale School of Medicine, which were stored in the Dryad database. The database is a publicly accessible data repository that authors have contributed to making their research data discoverable, freely reusable, and citable. For more details, this link can be referred: https://datadryad.org/stash/dataset/doi:10.5061/dryad.4f4qrfj95 (Su et al., 2021; Wilson, 2020).

### Inclusion Criteria

1) Age ≥18 years old; 2) patients with AKI, which was diagnosed based on the KDIGO (Kidney Disease: Improving Global Outcomes) AKI criterion.

### Exclusion Criteria

1) Previous dialysis patients; 2) end-stage renal disease; 3) initial serum creatinine ≥4.0 mg/L; 4) patients receiving hospice care; 5) patients undergoing kidney transplantation within 6 months.

### Diagnosis of Acute Kidney Injury

The diagnostic algorithm for AKI (KDIGO AKI criteria) had been incorporated into the epic electronic health record system. A “pop-up” warning for AKI based on the electronic health record, along with an accompanying AKI order, was set when the clinician opens the patient’s medical record. When the system was turned on, the algorithm collected the patient’s associated indices for the purpose of determining if the patient was complicated with AKI. This procedure was part of a system that automatically collects essential indications and generates alerts without human interaction, and the researchers at all participating hospitals were trained to guarantee that the system was implemented correctly and reliably.

### Participants

From 29 March 2018 to 14 December 2019, this research enrolled 6,030 individuals with AKI who satisfied the inclusion and exclusion criteria.

### Outcome Indicators Involved in This Study

The primary outcomes were AKI progression within 14 days, 14-day dialysis, discharge to home within 14 days, and 14-day mortality.

### AKI Progression

AKI progression was defined as a rise in the AKI stage within 14 days based on the KDIGO AKI criterion within 14 days.

### Collection of Clinical and Biochemical Data

1) Demographic data: age, sex, and ethnicity; 2) previous medical history: congestive heart failure, CKD, chronic obstructive pulmonary disease (COPD), diabetes, malignancy, and liver disease, as well as the complication index Elixhauser comorbidity score; 3) laboratory examination (when diagnosing AKI): anion gap, bicarbonate, BUN, chloridion, platelets, K^+^, and serum creatinine; 4) drug use: any diuretic within 24 h of AKI, NSAID treatment within 72 h before the onset of AKI, NSAIDs within 24 h before the onset of AKI, NSAIDs within the 24-h onset of AKI, PPI within 72 h before AKI, ACEi/ARB within 72 h before the onset of AKI, and fluid bolus within the 24-h onset of AKI; 5) other indicators: mean arterial pressure (MAP), SOFA score, nephrology consults, and contrast in prior 72 h; 6) related outcome indicators: 14-day mortality, AKI progression, and dialysis within 14 days. All of the aforementioned indicators were gathered prospectively, following the research protocol.

### Statistical Analysis

1) Statistical description: continuous variables were described by mean ± SD, while calculated data were reported using the number of cases and percentage. When comparing two groups, the *t*-test was used if the continuous variables had a normal distribution, and the variance was homogeneous; otherwise, the non-parametric test was used, and the chi-squared test was used for counting data. 2) The association between NSAIDs and AKI progression, 14-day dialysis, discharge to home, and 14-day mortality was evaluated using univariate and multivariate logistic regression analyses. 3) We conducted a subgroup analysis of age ≥ 65 years, CKD, congestive heart failure, hypertension, and liver disease. 4) Adjustment variables were chosen if they impacted the effect estimate of NSAIDs by more than 10% and were recognized in the literature, and we also eliminated those that were collinear. 5) EmpowerStats 2.0 (Copyright 2009 X&Y Solutions, Inc.) and R software were used to conduct all statistical analyses (3.4.3). *p* < 0.05 was statistically significant.

## Results

### Baseline Characteristics of Included Patients

Our study enrolled a total of 6,030 patients with AKI, the median of age was 69.32 67.00 ± 15.38 years, 3,148 (52.21%) patients were male, and the median of mean arterial pressure (MAP) was 84.82 ± 14.65 mmHg. The prevalence estimates of congestive heart failure, CKD, chronic obstructive pulmonary disease (COPD), diabetes, malignancy, and liver disease were 2,658 (44.08%), 2,290 (37.98%), 2,064 (34.23%), 2,290 (37.98%), 931 (15.44%), and 855 (14.18%), respectively. The NSAID treatments within 72 h before the onset of AKI, within 24 h before the onset of AKI, and within the 24-h onset of AKI were , respectively, 791 (13.12%), 600 (9.95%), and 310 (5.14%). The progression estimates of AKI within 14 days, 14-day dialysis, discharge to home, and 14-day mortality in patients with AKI, were, respectively, 948 (15.72%), 199(3.30%), 2,997 (49.70%), and 537 (8.91%) (Table 1).

**TABLE 1:** Baseline characteristics of included patients.

Variable	Mean ± SD/N (%)
Age, years	67.00 ± 15.38
Gender (M/F)	3,148 (52.21%)/2,882 (47.79%)
MAP, mmHg	84.82 ± 14.65
Ethnicity	
Hispanic, *n* (%)	620 (10.28%)
African American, *n* (%)	946 (15.69%)
Other, *n* (%)	4,154 (72.62%)
Congestive heart failure, *n* (%)	2,658 (44.08%)
CKD, *n* (%)	2,290 (37.98%)
COPD, *n* (%)	2064 (34.23%)
Diabetes, *n* (%)	2,290 (37.98%)
Malignancy, *n* (%)	931 (15.44%)
Liver disease, *n* (%)	855 (14.18%)
Elixhauser comorbidity score	6.32 ± 2.84
Hospital	
Urban teaching, *n* (%)	4,698 (77.91%)
Suburban non-teaching, *n* (%)	1,332 (22.09%)
Anion gap, mmol/L	12.40 ± 4.34
Bicarbonate, mmol/L	23.72 ± 5.23
BUN, mg/dL	32.05 ± 18.99
Serum creatinine, mg/dL	1.17 ± 0.66
Chloridion, mmol/L	102.04 ± 6.59
Na^+^, mmol/L	138.16 ± 5.27
Platelet, x1,000/μl	215.55 ± 108.08
SOFA score	2.45 ± 2.11
K^+^, mmol/L)	4.24 ± 0.64
eGFR, ml/min	60.87 ± 31.58
Alert, *n* (%)	653 (10.83%)
Any diuretic post 24 h of AKI, *n* (%)	1,674 (27.76%)
Nephrology consult, *n* (%)	1,437 (23.83%)
Contrast in prior 72 h, *n* (%)	217 (3.60%)
NSAIDs within 72 h before the onset of AKI, *n* (%)	791 (13.12%)
NSAIDs within 24 h before the onset of AKI, *n* (%)	600 (9.95%)
NSAIDs within the 24-h onset of AKI, *n* (%)	310 (5.14%)
PPI within 72 h before the onset of AKI, *n* (%)	1,361 (22.57%)
ACEi/ARB within 72 h before onset of AKI, *n* (%)	1,093 (18.13%)
Fluid bolus post 24 h-onset of AKI, *n* (%)	1,443 (23.93%)
AKI progression AKI within 14 days, *n* (%)	948 (15.72%)
14-day dialysis, *n* (%)	199 (3.30%)
Discharge to home, *n* (%)	2,997 (49.70%)
14-day mortality, *n* (%)	537 (8.91%)

### The Relationship Between NSAIDs and AKI Progression Within 14 Days in Patients With AKI

Univariate analysis revealed that NSAIDs within 72 h before the onset of AKI and NSAIDs within 24 h before the onset of AKI might significantly lower the AKI progression, and the OR values were 0.72 (95% CI: 0.58–0.90, *p* = 0.004) and 0.76 (95% CI: 0.59–0.97, *p* = 0.031). After adjusting the related confounding variables, it was determined that NSAID treatment 72 h before the onset of AKI and 24 h before the onset of AKI was not associated with the AKI progression. However, NSAID treatments within the 24-h onset of AKI could develop AKI progression; their adjusted OR values were, respectively, 1.04 (95% CI: 0.81–1.35, *p* = 0.746), 1.11 (95% CI: 0.83–1.48, *p* = 0.485), and 1.50 (95% CI: 1.02–2.19, *p* = 0.037). The subgroup analysis showed that in addition to the presence of CKD, NSAID treatments within 72 h before the onset of AKI and NSAID treatments within 24 h before the onset of AKI promoted AKI progression, while other conditions did not promote renal disease progressions, such as whether the age ≥ 65 years or age < 65 years, and whether it was accompanied by congestive heart failure, hypertension, and liver disease. However, NSAID treatment within the 24-h onset of AKI could significantly promote AKI progression in patients with CKD, congestive heart failure, hypertension, liver disease, and alert; their OR values were, respectively, 7.57 (95% CI: 3.02–18.99, *p* < 0.001), 4.31 (95% CI: 2.12–8.75, *p* < 0.001), 1.65 (95% CI: 1.05–2.59, *p* = 0.03) 3.22 (95% CI: 1.35–7.71, *p* = 0.009), and 2.23 (95% CI: 1.34–3.74, *p* = 0.002) (Tables 2, 3).

**TABLE 2:** Relationship between NSAIDs and AKI progression and the prognosis of patients with AKI.

Exposure	Non-adjusted	*p*-value	Adjusted	*p*-value
AKI progression within 14 days				
NSAIDs within 72 h before the onset of AKI				
No	Reference		Reference	
Yes	0.72 (0.58, 0.90)	0.004	1.04 (0.81, 1.35)	0.746
NSAIDs within 24 h before the onset of AKI				
No	Reference		Reference	
Yes	0.76 (0.59, 0.97)	0.031	1.11 (0.83, 1.48)	0.485
NSAIDs within the 24-h onset of AKI				
No	Reference		Reference	
Yes	0.81 (0.58, 1.13)	0.216	1.50 (1.02, 2.19)	0.037
14-day dialysis				
NSAIDs within 72 h before the onset of AKI				
No	Reference		Reference	
Yes	0.49 (0.28, 0.85)	0.011	1.35 (0.69, 2.66)	0.384
NSAIDs within 24 h before the onset of AKI				
No	Reference		Reference	
Yes	0.52 (0.28, 0.96)	0.037	1.56 (0.71, 3.42)	0.263
NSAIDs within the 24-h onset of AKI				
No	Reference		Reference	
Yes	0.57 (0.25, 1.28)	0.173	4.20 (1.47, 11.97)	0.007
Discharge to home				
NSAIDs within 72 h before the onset of AKI				
No	Reference		Reference	
Yes	1.46 (1.26, 1.70)	<0.001	0.97 (0.82, 1.16)	0.775
NSAIDs within 24 h before the onset of AKI				
No	Reference		Reference	
Yes	1.49 (1.26, 1.77)	<0.001	1.04 (0.85, 1.27)	0.712
NSAIDs within the 24-h onset of AKI				
No	Reference		Reference	
Yes	1.19 (0.95, 1.50)	0.132	0.71 (0.54, 0.92)	0.011
14-day mortality				
NSAIDs within 72 h before the onset of AKI				
No	Reference		Reference	
Yes	0.31 (0.21, 0.47)	<0.001	0.83 (0.42, 1.64)	0.589
NSAIDs within 24 h before the onset of AKI				
No	Reference		Reference	
Yes	0.35 (0.22, 0.54)	<0.001	0.52 (0.33, 0.82)	0.005
NSAIDs within the 24 h-onset of AKI				
No	Reference		Reference	
Yes	0.40 (0.22, 0.71)	0.002	0.83 (0.42, 1.64)	0.589

Adjusted variables: age; race; gender; Elixhauser comorbidity score; hospital; any diuretic after the 24-h onset of AKI; CKD (chronic kidney disease); alert; anion gap at rand; bicarbonate; K^+^; chloridion; Na^+^; PPI (proton pump inhibitors) within 72 h before the onset of AKI; fluid bolus after the 24-h onset of AKI; aminoglycoside before the 24-h onset of AKI; ACEi/ARB within the 72-h onset of AKI; SOFA score; nephrology.

**TABLE 3:** Relationship between NSAIDs and AKI progression in patients with AKI.

Exposure	Non-adjusted	*p*-value	Adjusted	*p*-value
Age < 65 years				
NSAIDs within 72 h before the onset of AKI	0.69 (0.50, 0.93)	0.017	1.20 (0.84, 1.72)	0.319
NSAIDs within 24 h before the onset of AKI	0.71 (0.51, 1.01)	0.055	1.20 (0.81, 1.79)	0.361
NSAIDs within the 24-h onset of AKI	0.71 (0.45, 1.12)	0.138	1.33 (0.80, 2.23)	0.276
Age ≥ 65 years				
NSAIDs within 72 h before the onset of AKI	0.69 (0.49, 0.96)	0.029	0.91 (0.62, 1.33)	0.613
NSAIDs within 24 h before the onset of AKI	0.72 (0.49, 1.05)	0.091	1.05 (0.68, 1.61)	0.834
NSAIDs within the 24-h onset of AKI	0.88 (0.53, 1.45)	0.613	1.71 (0.97, 3.02)	0.065
Without CKD				
NSAIDs within 72 h before the onset of AKI	0.55 (0.42, 0.72)	<0.001	0.83 (0.61, 1.13)	0.234
NSAIDs within 24 h before the onset of AKI	0.58 (0.43, 0.78)	<0.001	0.87 (0.62, 1.21)	0.404
NSAIDs within the 24-h onset of AKI	0.61 (0.41, 0.90)	0.013	1.08 (0.70, 1.67)	0.723
With CKD				
NSAIDs within 72 h before the onset of AKI	1.59 (1.04, 2.42)	0.032	2.04 (1.20, 3.46)	0.008
NSAIDs within 24 h before the onset of AKI	1.81 (1.12, 2.94)	0.016	2.59 (1.40, 4.82)	0.003
NSAIDs within the 24-h onset of AKI	3.14 (1.49, 6.61)	0.003	7.57 (3.02, 18.99)	<0.001
Without congestive heart failure				
NSAIDs within 72 h before the onset of AKI	0.62 (0.48, 0.82)	<0.001	0.96 (0.70, 1.31)	0.791
NSAIDs within 24 h before the onset of AKI	0.64 (0.47, 0.86)	0.003	0.98 (0.70, 1.39)	0.929
NSAIDs within the 24-h onset of AKI	0.55 (0.36, 0.83)	0.005	1.02 (0.63, 1.64)	0.944
With congestive heart failure				
NSAIDs within 72 h before the onset of AKI	1.08 (0.71, 1.66)	0.711	1.32 (0.81, 2.15)	0.260
NSAIDs within 24 h before the onset of AKI	1.27 (0.79, 2.05)	0.324	1.60 (0.93, 2.75)	0.090
NSAIDs within the 24-h onset of AKI	2.81 (1.52, 5.19)	<0.001	4.31 (2.12, 8.75)	<0.001
Without hypertension				
NSAIDs within 72 h before the onset of AKI	0.50 (0.32, 0.78)	0.003	1.04 (0.61, 1.76) 1	0.888
NSAIDs within 24 h before the onset of AKI	0.51 (0.31, 0.84)	0.008	1.10 (0.62, 1.97)	0.740
NSAIDs within the 24-h onset of AKI	0.50 (0.26, 0.96)	0.036	1.45 (0.69, 3.06)	0.329
With hypertension				
NSAIDs within 72 h before the onset of AKI	0.79 (0.61, 1.02)	0.070	1.06 (0.78, 1.43)	0.711
NSAIDs within 24 h before the onset of AKI	0.84 (0.63, 1.12)	0.241	1.15 (0.82, 1.62)	0.419
NSAIDs within the 24-h onset of AKI	0.93 (0.63, 1.39)	0.737	1.65 (1.05, 2.59)	0.030
Without liver disease				
NSAIDs within 72 h before the onset of AKI	0.80 (0.62, 1.03)	0.083	1.07 (0.81, 1.43)	0.623
NSAIDs within 24 h before the onset of AKI	0.89 (0.67, 1.17)	0.387	1.20 (0.87, 1.65)	0.259
NSAIDs within the 24-h onset of AKI	0.78 (0.52, 1.15)	0.205	1.33 (0.86, 2.06)	0.198
With liver disease				
NSAIDs within 72 h before the onset of AKI	0.60 (0.36, 1.02)	0.058	0.88 (0.47, 1.66)	0.704
NSAIDs within 24 h before the onset of AKI	0.50 (0.27, 0.93)	0.029	0.75 (0.36, 1.55)	0.435
NSAIDs within the 24-h onset of AKI	1.20 (0.58, 2.48)	0.620	3.22 (1.35, 7.71)	0.009
Without alert (usual care)				
NSAIDs within 72 h before the onset of AKI	0.74 (0.54, 1.02)	0.065	1.12 (0.78, 1.60)	0.551
NSAIDs within 24 h before the onset of AKI	0.81 (0.57, 1.14)	0.221	1.21 (0.81, 1.80)	0.347
NSAIDs within the 24-h onset of AKI	0.57 (0.33, 0.96)	0.034	1.01 (0.56, 1.83)	0.962
With alert				
NSAIDs within 72 h before the onset of AKI	0.70 (0.51, 0.96)	0.029	0.96 (0.66, 1.40)	0.821
NSAIDs within 24 h before the onset of AKI	0.71 (0.49, 1.03)	0.068	1.02 (0.66, 1.58)	0.924
NSAIDs within the 24-h onset of AKI	1.12 (0.72, 1.74)	0.628	2.23 (1.34, 3.74)	0.002

Adjusted variables (without the subgroup analysis variables themselves): age; race; gender; Elixhauser comorbidity score; hospital; any diuretic after the 24-h onset of AKI; CKD (chronic kidney disease); alert; anion gap at rand; bicarbonate; K+; chloridion; Na+; PPI (proton pump inhibitors) within 72 h before the onset of AKI; fluid bolus after the 24-h onset of AKI; aminoglycoside before the 24-h onset of AKI; ACEi/ARB within the 72-h onset of AKI; SOFA score; nephrology.

### The Relationship Between NSAIDs and 14-Day Dialysis in Patients With AKI

Univariate analysis showed that NSAID treatments within 72 h before the onset of AKI and NSAID treatments within 24 h before the onset of AKI could reduce the 14-day dialysis, their OR values were, respectively, 0.49 (95% CI: 0.28–0.85, *p* = 0.011) and 0.52 (95% CI: 0.28–0.96, *p* = 0.037) and that NSAIDs within the 24-h onset of AKI were not associated with the 14-day dialysis; the OR value was 0.57 (95% CI: 0.25–1.28, *p* = 0.173). After adjusting the related confounding variables, only NSAIDs within the 24-h onset of AKI were associated with the 14-day dialysis, and the adjusted OR was 4.20 (95% CI: 1.47–11.97, *p* = 0.007). The subgroup analysis revealed that when AKI patients were co-occurring with the following conditions, age ≥ 65 years, CKD, congestive heart failure, hypertension, liver disease, and alert, NSAIDs within the 24-h onset of AKI significantly improved the 14-day dialysis and that in patients with liver disease, NSAIDs within 24 h before the onset of AKI also significantly improved the 14-day dialysis (*p* < 0.05) (Tables 2, 4).

**TABLE 4:** Relationship between NSAIDs and 14-day dialysis in patients with AKI.

Exposure	Non-adjusted	*p*-value	Adjusted	*p*-value
Age < 65 years				
NSAIDs within 72 h before the onset of AKI	0.34 (0.15, 0.78)	0.011	1.22 (0.37, 4.06)	0.742
NSAIDs within 24 h before the onset of AKI	0.38 (0.15, 0.95)	0.039	1.15 (0.30, 4.46)	0.837
NSAIDs within the 24-h onset of AKI	0.29 (0.07, 1.18)	0.083	1.39 (0.23, 8.27)	0.716
Age ≥ 65 years				
NSAIDs within 72 h before the onset of AKI	0.61 (0.29, 1.26)	0.179	1.76 (0.73, 4.23)	0.205
NSAIDs within 24 h before the onset of AKI	0.61 (0.27, 1.40)	0.244	2.58 (0.91, 7.30)	0.073
NSAIDs within the 24-h onset of AKI	0.88 (0.32, 2.42)	0.800	8.09 (1.90, 34.35)	0.005
Without CKD				
NSAIDs within 72 h before the onset of AKI	0.39 (0.18, 0.85)	0.018	1.46 (0.54, 3.92)	0.456
NSAIDs within 24 h before the onset of AKI	0.54 (0.25, 1.16)	0.115	2.17 (0.78, 6.04)	0.138
NSAIDs within the 24-h onset of AKI	0.42 (0.13, 1.34)	0.144	2.79 (0.66, 11.80)	0.164
With CKD				
NSAIDs within 72 h before the onset of AKI	1.07 (0.49, 2.34)	0.875	1.33 (0.48, 3.64)	0.584
NSAIDs within 24 h before the onset of AKI	0.87 (0.31, 2.40)	0.781	1.23 (0.31, 4.80)	0.769
NSAIDs within the 24-h onset of AKI	2.18 (0.65, 7.27)	0.207	15.19 (2.57, 89.84)	0.003
Without congestive heart failure				
NSAIDs within 72 h before the onset of AKI	0.36 (0.17, 0.78)	0.010	1.07 (0.40, 2.85)	0.897
NSAIDs within 24 h before the onset of AKI	0.34 (0.14, 0.85)	0.021	1.00 (0.30, 3.29)	0.997
NSAIDs within the 24-h onset of AKI	0.13 (0.02, 0.93)	0.043	0.95 (0.11, 8.47)	0.966
With congestive heart failure				
NSAIDs within 72 h before the onset of AKI	1.07 (0.49, 2.35)	0.857	2.20 (0.77, 6.24)	0.140
NSAIDs within 24 h before the onset of AKI	1.30 (0.56, 3.04)	0.538	3.93 (1.18, 13.11)	0.026
NSAIDs within the 24-h onset of AKI	2.87 (1.11, 7.40)	0.029	18.39 (4.08, 82.89)	<0.001
Without hypertension				
NSAIDs within 72 h before the onset of AKI	0.33 (0.10, 1.08)	0.067	1.52 (0.22, 10.51)	0.670
NSAIDs within 24 h before the onset of AKI	0.14 (0.02, 1.04)	0.055	0.32 (0.02, 6.20)	0.449
NSAIDs within the 24-h onset of AKI	0.00 (0.00, Inf)	0.981	0.00 (0.00, Inf)	0.998
With hypertension				
NSAIDs within 72 h before the onset of AKI	0.55 (0.30, 1.02)	0.057	1.30 (0.59, 2.85)	0.510
NSAIDs within 24 h before the onset of AKI	0.68 (0.36, 1.30)	0.246	1.94 (0.82, 4.60)	0.133
NSAIDs within the 24-h onset of AKI	0.87 (0.38, 1.98)	0.736	5.34 (1.79, 15.96)	0.003
Without liver disease				
NSAIDs within 72 h before the onset of AKI	0.17 (0.04, 0.72)	0.015	0.37 (0.06, 2.37)	0.297
NSAIDs within 24 h before the onset of AKI	0.12 (0.02, 0.84)	0.033	0.18 (0.01, 2.84)	0.221
NSAIDs within the 24-h onset of AKI	0.51 (0.12, 2.15)	0.356	5.16 (0.71, 37.70)	0.106
With liver disease				
NSAIDs within 72 h before the onset of AKI	0.82 (0.45, 1.51)	0.532	2.08 (0.98, 4.44)	0.058
NSAIDs within 24 h before the onset of AKI	0.92 (0.48, 1.78)	0.807	2.83 (1.20, 6.68)	0.018
NSAIDs within the 24-h onset of AKI	0.70 (0.26, 1.92)	0.493	4.18 (1.12, 15.57)	0.033
Without alert (usual care)				
NSAIDs within 72 h before the onset of AKI	0.51 (0.23, 1.11)	0.09	1.34 (0.51, 3.51)	0.545
NSAIDs within 24 h before the onset of AKI	0.58 (0.25, 1.34)	0.200	1.56 (0.54, 4.45)	0.410
NSAIDs within the 24-h onset of AKI	0.56 (0.17, 1.77)	0.321	3.19 (0.64, 15.80)	0.155
With alert				
NSAIDs within 72 h before the onset of AKI	0.48 (0.22, 1.04)	0.061	1.03 (0.36, 2.98)	0.952
NSAIDs within 24 h before the onset of AKI	0.47 (0.19, 1.16)	0.100	1.03 (0.27, 3.88)	0.969
NSAIDs within the 24-h onset of AKI	0.58 (0.18, 1.85)	0.359	6.23 (1.42, 27.22)	0.015

Adjusted variables (without the subgroup analysis variables themselves): age; race; gender; Elixhauser comorbidity score; hospital; any diuretic after the 24-h onset of AKI; CKD (chronic kidney disease); alert; anion gap at rand; bicarbonate; K+; chloridion; Na+; PPI (proton pump inhibitors) within 72 h before the onset of AKI; fluid bolus after the 24-h onset of AKI; aminoglycoside before the 24-h onset of AKI; ACEi/ARB within the 72-h onset of AKI; SOFA score; nephrology.

### The Relationship Between NSAIDs and the Discharge to Home in Patients With AKI

Univariate analysis showed that NSAID treatments within 72 h before the onset of AKI and NSAID treatments within 24 h before the onset of AKI could increase the discharge to home, and their ORs were, respectively, 1.46 (95% CI: 1.26–1.70, *p* < 0.001) and 1.49 (95% CI: 1.26–1.77, *p* < 0.001) and that NSAIDs within the 24-h onset of AKI were not associated with the discharge to home, and the OR value was 1.19 (95% CI: 0.95–1.50, *p* = 0.132). After adjusting the related confounding variables, only NSAIDs within the 24-h onset of AKI decreased the discharge to home, and the adjusted OR was 0.71 (95% CI: 0.54–0.92, *p* = 0.011). The subgroup analysis revealed that when AKI patients were co-occurring with the following conditions, age ≥ 65 years, CKD, congestive heart failure, hypertension, liver disease, and whether with or without, alert, NSAIDs within the 24-h onset of AKI significantly reduced the discharge to home (*p* < 0.05) (Tables 2, 5).

**TABLE 5:** Relationship between NSAIDs and the discharge to home in patients with AKI.

Exposure	Non-adjusted	*p*-value	Adjusted	*p*-value
Age < 65 years				
NSAIDs within 72 h before the onset of AKI	1.71 (1.34, 2.19)	<0.001	1.11 (0.84, 1.48)	0.460
NSAIDs within 24 h before the onset of AKI	1.76 (1.34, 2.32)	<0.001	1.18 (0.86, 1.62)	0.305
NSAIDs within the 24-h onset of AKI	1.75 (1.21, 2.51)	0.003	1.21 (0.80, 1.83)	0.372
Age ≥ 65 years				
NSAIDs within 72 h before the onset of AKI	1.10 (0.89, 1.36)	0.368	0.94 (0.75, 1.19)	0.607
NSAIDs within 24 h before the onset of AKI	1.14 (0.90, 1.46)	0.271	1.01 (0.78, 1.32)	0.925
NSAIDs within the 24-h onset of AKI	0.70 (0.49, 0.99)	0.046	0.53 (0.36, 0.77)	<0.001
Without CKD				
NSAIDs within 72 h before the onset of AKI	1.66 (1.40, 1.97)	<0.001	1.09 (0.89, 1.34)	0.392
NSAIDs within 24 h before the onset of AKI	1.63 (1.35, 1.98)	<0.001	1.13 (0.90, 1.41)	0.295
NSAIDs within the 24-h onset of AKI	1.24 (0.97, 1.59)	0.086	0.78 (0.58, 1.04)	0.094
With CKD				
NSAIDs within 72 h before the onset of AKI	0.72 (0.51, 1.02)	0.064	0.58 (0.38, 0.89)	0.012
NSAIDs within 24 h before the onset of AKI	0.78 (0.51, 1.18)	0.242	0.68 (0.42, 1.11)	0.126
NSAIDs within the 24-h onset of AKI	0.47 (0.21, 1.02)	0.056	0.33 (0.14, 0.80)	0.014
Without congestive heart failure				
NSAIDs within 72 h before the onset of AKI	1.46 (1.23, 1.75)	<0.001	1.08 (0.87, 1.32)	0.491
NSAIDs within 24 h before the onset of AKI	1.47 (1.20, 1.78)	<0.001	1.09 (0.87, 1.38)	0.452
NSAIDs within the 24-h onset of AKI	1.20 (0.93, 1.54)	0.167	0.80 (0.59, 1.08)	0.148
With congestive heart failure				
NSAIDs within 72 h before the onset of AKI	0.96 (0.69, 1.32)	0.792	0.78 (0.54, 1.14)	0.199
NSAIDs within 24 h before the onset of AKI	1.01 (0.69, 1.47)	0.962	0.94 (0.61, 1.45)	0.796
NSAIDs within the 24-h onset of AKI	0.53 (0.28, 1.00)	0.049	0.40 (0.20, 0.80)	0.010
Without hypertension				
NSAIDs within 72 h before the onset of AKI	2.11 (1.52, 2.93)	<0.001	1.20 (0.80, 1.80)	0.383
NSAIDs within 24 h before the onset of AKI	2.11 (1.46, 3.04)	<0.001	1.20 (0.77, 1.88)	0.422
NSAIDs within the 24-h onset of AKI	2.49 (1.54, 4.01)	<0.001	1.37 (0.77, 2.46)	0.288
With hypertension				
NSAIDs within 72 h before the onset of AKI	1.26 (1.06, 1.50)	0.009	0.92 (0.75, 1.13)	0.418
NSAIDs within 24 h before the onset of AKI	1.29 (1.06, 1.57)	0.010	0.98 (0.78, 1.23)	0.834
NSAIDs within the 24-h onset of AKI	0.84 (0.64, 1.11)	0.232	0.56 (0.41, 0.77)	<0.004
Without liver disease				
NSAIDs within 72 h before the onset of AKI	1.54 (0.99, 2.41)	0.058	0.93 (0.54, 1.60)	0.787
NSAIDs within 24 h before the onset of AKI	1.77 (1.07, 2.94)	0.027	1.11 (0.60, 2.05)	0.751
NSAIDs within the 24-h onset of AKI	1.30 (0.65, 2.62)	0.461	0.63 (0.26, 1.50)	0.293
With liver disease				
NSAIDs within 72 h before the onset of AKI	1.43 (1.21, 1.68)	<0.001	0.98 (0.81, 1.19)	0.842
NSAIDs within 24 h before the onset of AKI	1.44 (1.20, 1.72)	<0.001	1.03 (0.83, 1.28)	0.758
NSAIDs within the 24-h onset of AKI	1.16 (0.91, 1.48)	0.238	0.72 (0.54, 0.95)	0.022
Without alert (usual care)				
NSAIDs within 72 h before the onset of AKI	1.54 (1.25, 1.91)	<0.001	0.95 (0.73, 1.22)	0.672
NSAIDs within 24 h before the onset of AKI	1.55 (1.22, 1.97)	<0.001	0.98 (0.74, 1.30)	0.875
NSAIDs within the 24-h onset of AKI	1.38 (1.01, 1.89)	0.046	0.68 (0.47, 0.99)	0.043
With alert				
NSAIDs within 72 h before the onset of AKI	1.38 (1.12, 1.71)	<0.001	0.93 (0.73, 1.20)	0.597
NSAIDs within 24 h before the onset of AKI	1.44 (1.12, 1.84)	0.004	1.00 (0.75, 1.33)	0.999
NSAIDs within the 24-h onset of AKI	1.01 (0.72, 1.41)	0.966	0.64 (0.43, 0.95)	0.028

Adjusted variables (without the subgroup analysis variables themselves): age; race; gender; Elixhauser comorbidity score; hospital; any diuretic after the 24-h onset of AKI; CKD (chronic kidney disease); alert; anion gap at rand; bicarbonate; K+; chloridion; Na+; PPI (proton pump inhibitors) within 72 h before the onset of AKI; fluid bolus after the 24-h onset of AKI; aminoglycoside before the 24-h onset of AKI; ACEi/ARB within the 72-h onset of AKI; SOFA score; nephrology.

### The Relationship Between NSAIDs and Death Within 14 Days in Patients With AKI

Univariate analysis showed that NSAID treatments within 72 h before the onset of AKI, NSAID treatments within 24 h before the onset of AKI, and NSAID treatments within the 24-h onset of AKI could decrease the 14-day mortality; their ORs were, respectively, 0.31 (95% CI: 0.21–0.47, *p* < 0.001), 0.35 (95% CI: 0.22–0.54, *p* < 0.001), and 0.40 (95% CI: 0.22–0.71, *p* = 0.002). After adjusting the related confounding variables, only NSAIDs within 24 h before the onset of AKI could reduce the death within 14 days, and the OR value was 0.52 (95% CI: 0.33–0.82, *p* = 0.005). The subgroup analysis revealed that when AKI patients were complicated with the following conditions, age ≥ 65 years, congestive heart failure, hypertension, and alert, NSAIDs within 72 h before the onset of AKI significantly reduced the 14-day mortality and that in patients with congestive heart failure, NSAIDs within 24 h before the onset of AKI would also significantly reduce the 14-day mortality and that in patients with the liver disease, NSAID treatments within the 24-h onset of AKI could increase the 14-day mortality (Tables 2, 6).

**TABLE 6:** Relationship between NSAIDs and the 14-day mortality in patients with AKI.

Exposure	Non-adjusted	*p*-value	Adjusted	*p*-value
Age < 65 years				
NSAIDs within 72 h before the onset of AKI	0.25 (0.12, 0.52)	<0.001	0.54 (0.24, 1.22)	0.136
NSAIDs within 24 h before the onset of AKI	0.26 (0.11, 0.59)	0.001	0.49 (0.19, 1.26)	0.137
NSAIDs within the 24-h onset of AKI	0.33 (0.12, 0.91)	0.033	0.80 (0.26, 2.44)	0.697
Age ≥ 65 years				
NSAIDs within 72 h before the onset of AKI	0.37 (0.22, 0.62)	<0.001	0.50 (0.28, 0.89)	0.017
NSAIDs within 24 h before the onset of AKI	0.40 (0.22, 0.72)	0.002	0.54 (0.29, 1.03)	0.061
NSAIDs within the 24-h onset of AKI	0.43 (0.19, 0.98)	0.044	0.83 (0.35, 1.99)	0.673
Without CKD				
NSAIDs within 72 h before the onset of AKI	0.26 (0.17, 0.42)	<0.001	0.57 (0.34, 0.97)	0.036
NSAIDs within 24 h before the onset of AKI	0.28 (0.17, 0.47)	<0.001	0.58 (0.32, 1.05)	0.072
NSAIDs within the 24-h onset of AKI	0.27 (0.13, 0.55)	<0.001	0.68 (0.31, 1.53)	0.355
With CKD				
NSAIDs within 72 h before the onset of AKI	0.49 (0.21, 1.13)	0.094	0.35 (0.12, 1.02)	0.053
NSAIDs within 24 h before the onset of AKI	0.61 (0.24, 1.51)	0.285	0.36 (0.10, 1.26)	0.109
NSAIDs within the 24-h onset of AKI	1.68 (0.58, 4.86)	0.338	1.58 (0.39, 6.44)	0.520
Without congestive heart failure				
NSAIDs within 72 h before the onset of AKI	0.33 (0.21, 0.52)	<0.001	0.58 (0.34, 1.01)	0.056
NSAIDs within 24 h before the onset of AKI	0.38 (0.23, 0.62)	<0.001	0.67 (0.36, 1.22)	0.187
NSAIDs within the 24-h onset of AKI	0.25 (0.11, 0.57)	0.001	0.58 (0.22, 1.50)	0.262
With congestive heart failure				
NSAIDs within 72 h before the onset of AKI	0.33 (0.15, 0.76)	0.009	0.35 (0.13, 0.90)	0,030
NSAIDs within 24 h before the onset of AKI	0.31 (0.11, 0.85)	0.023	0.31 (0.10, 0.95)	0.040
NSAIDs within the 24-h onset of AKI	1.32 (0.55, 3.13)	0.533	1.71 (0.59, 4.91)	0.322
Without hypertension				
NSAIDs within 72 h before the onset of AKI	0.21 (0.10, 0.46)	<0.001	0.65 (0.26, 1.61)	0.349
NSAIDs within 24 h before the onset of AKI	0.20 (0.08, 0.50)	<0.001	0.61 (0.21, 1.74)	0.356
NSAIDs within the 24-h onset of AKI	0.07 (0.01, 0.50)	0.008	0.34 (0.04, 2.72)	0.309
With hypertension				
NSAIDs within 72 h before the onset of AKI	0.35 (0.22, 0.55)	<0.001	0.49 (0.28, 0.85)	0.012
NSAIDs within 24 h before the onset of AKI	0.40 (0.24, 0.66)	<0.001	0.54 (0.29, 1.01)	0.052
NSAIDs within the 24-h onset of AKI	0.60 (0.32, 1.11)	0.101	1.14 (0.55, 2.38)	0.720
Without liver disease				
NSAIDs within 72 h before the onset of AKI	0.36 (0.24, 0.56)	<0.001	0.55 (0.33, 0.93)	0.024
NSAIDs within 24 h before the onset of AKI	0.43 (0.27, 0.69)	<0.001	0.62 (0.35, 1.10)	0.103
NSAIDs within the 24-h onset of AKI	0.26 (0.12, 0.60)	0.001	0.50 (0.20, 1.27)	0.144
With liver disease				
NSAIDs within 72 h before the onset of AKI	0.20 (0.07, 0.57)	0.002	0.35 (0.11, 1.12)	0.078
NSAIDs within 24 h before the onset of AKI	0.14 (0.03, 0.56)	0.006	0.22 (0.04, 1.06)	0.060
NSAIDs within the 24-h onset of AKI	1.03 (0.42, 2.54)	0.951	3.52 (1.02, 12.18)	0.047
Without alert (usual care)				
NSAIDs within 72 h before the onset of AKI	0.33 (0.19, 0.58)	<0.001	0.54 (0.29, 1.02)	0.056
NSAIDs within 24 h before the onset of AKI	0.35 (0.19, 0.64)	<0.001	0.53 (0.26, 1.09)	0.086
NSAIDs within the 24-h onset of AKI	0.24 (0.09, 0.65)	0.005	0.60 (0.21, 1.73)	0.345
With alert				
NSAIDs within 72 h before the onset of AKI	0.30 (0.16, 0.53)	<0.001	0.46 (0.23, 0.93)	0.030
NSAIDs within 24 h before the onset of AKI	0.34 (0.18, 0.66)	0.001	0.50 (0.23, 1.08)	0.076
NSAIDs within the 24-h onset of AKI	0.59 (0.29, 1.22)	0.154	1.14 (0.47, 2.78)	0,772

Adjusted variables (without the subgroup analysis variables themselves): age; race; gender; Elixhauser comorbidity score; hospital; any diuretic after the 24-h onset of AKI; CKD (chronic kidney disease); alert; anion gap at rand; bicarbonate; K+; chloridion; Na+; PPI (proton pump inhibitors) within 72 h before the onset of AKI; fluid bolus after the 24-h onset of AKI; aminoglycoside before the 24-h onset of AKI; ACEi/ARB within the 72-h onset of AKI; SOFA score; nephrology.

## Discussion

This study discovered that taking NSAIDs within 72 and 24 h before the onset of AKI had no association with AKI progression, 14-day dialysis, and discharge to home in patients with AKI. In comparison, only NSAID treatment within the 24-h onset of AKI would promote AKI progression and worsen patient prognosis, especially in patients with age ≥ 65 years, CKD, congestive heart failure, hypertension, liver disease, and alert.

Previous research has established that NSAIDs increase the AKI risk and that patients with AKI have a considerably poorer prognosis. NSAIDs were usually considered a risk factor for triggering AKI, and healthcare workers have been advised to use NSAID medications with caution. However, the reality is that NSAIDs are one of the most commonly used drugs in the clinic and lack effective alternatives, and the standard use of NSAIDs cautiously lacks operability. This study found that the use of NSAIDs before the disease progressed to AKI did not promote the deterioration of renal function, increase dialysis rates, affect discharge to home, and increase mortality in AKI patients. In contrast, NSAIDs in AKI, even if used at the early stage of AKI, would lead to AKI progression and worsen the prognosis of patients with AKI. In a retrospective study of 2,340 people admitted to the ICU due to fractures, who had taken one or more doses of NSAIDs before 48 h of hospitalization, NSAID exposure was not associated with increased AKI progression, decreased AKI improvement, prolonged duration, or increased mortality (Hatton et al., 2020). Another systematic review and meta-analysis showed that perioperative NSAID treatment might be associated with increased disease-free and overall survival after cancer surgery (Shaji et al., 2021). The aforementioned two studies showed that the treatment of NSAIDs in either the early stage of AKI or perioperative periods did not confer adverse outcomes, which was consistent with the conclusions of our study.

Previous studies had shown that the use of NSAIDs in patients with AKI could significantly worsen the disease progression and prognosis (Su et al., 2021). However, our study found that even within the 24-h onset of AKI, 5.14% of patients continued to use NSAIDs and that NSAID treatments within the 24-h onset of AKI promoted AKI progression and 14-day dialysis and reduced the discharge to home. However, our study showed that NSAID treatments within the 24-h onset of AKI did not increase the 14-day mortality. The possible reasons are as follows: 1) first of all, the population involved in this study was different from the previous study population; the previous study population was the population without AKI, to explore whether the use of NSAIDs could induce AKI and worsen the prognosis of patients, while our study explored the effect of the use of NSAIDs on AKI progression and the prognosis of patients based on the fact that the patient’s disease had progressed to AKI. 2) The death outcome of our study was 14-day mortality. Because AKI due to NSAIDs was time- and dose-dependent (Zhang et al., 2017; Lucas et al., 2019), it might be that the dose and temporal effects of NSAIDs had not yet been fully realized, which resulted in a negative result.

Bruce Guthrie discovered that the incidence of AKI caused by NSAIDs was substantially greater in older patients than that in non-elderly individuals in their meta-analysis and systematic evaluation research, and the OR value was 2.51 (95% CI: 1.52–2.68) (Zhang et al., 2017). Kurina’s retrospective study also discovered that the older patients taking NSAIDs have greater risk of AKI (Nelson et al., 2019). Randomized controlled research on the metabolism of NSAIDs discovered that the metabolic rate of NSAIDs dropped considerably with age and that the degree of metabolic rate reduction was strongly related to the age-related loss in the renal function (McKeand et al., 2018). Pharmacologically, NSAIDs have a dose-dependent influence on the severity and risk of AKI. With increasing age, NSAID metabolism slowed, and NSAIDs accumulated in the body; this might be the reason why the elderly’s AKI progression and dialysis with 14 days rose obviously.

In addition, this study discovered that in patients with CKD, NSAIDs promoted AKI progression and decreased discharge to home in patients with AKI. Patients with CKD were more prone to accumulate NSAIDs and further impair kidney function due to their kidney dysfunction (Wu et al., 2015; Wolfe et al., 2021).

Congestive heart failure exacerbates cardiac ejection dysfunction, and hypertension can result in glomerular focal sphericity and local segmental sclerosis, which contribute to renal hypoperfusion (Dalal et al., 2022; Joslin et al., 2022). NSAIDs might reduce prostaglandin production, resulting in renal blood vessel constriction and decreased renal blood flow (Bindu et al., 2020). As a result, using NSAIDs in individuals with congestive heart failure and hypertension might exacerbate renal ischemia and damage. In addition, related studies demonstrated that patients with pre-renal parenchymal damage such as dehydration and hypovolemia and that even short-term NSAID usage might result in renal failure (Makris and Spanou, 2016), which corroborates the preceding assertion.

The use of NSAIDs in patients with liver disorders was observed to promote AKI progression and dialysis within 14 days and decrease the discharge to home in patients with AKI. NSAIDs might disrupt the intestinal barrier, resulting in bacterial translocation and toxic properties entering the liver and causing liver injury or inflammation (Licata et al., 2017; Utzeri and Usai, 2017). The liver is the primary site of NSAID metabolism (Ghlichloo and Gerriets, 2021). When a liver injury occurs, the metabolic rate of NSAIDs in the liver will inevitably decrease, resulting in NSAID buildup in the body and subsequent induction or aggravation of kidney damage. This might be the reason why NSAIDs promote the deterioration of renal function in patients with liver diseases.

This study found that taking NSAIDs in the early stages of AKI had a significant impact on the prognosis of patients with alertness of AKI but had no effect on the prognosis of patients receiving standard therapy. In our opinion, the reason why a patient with AKI was still receiving NSAID medications was that the patient might have additional conditions that continually needed the use of NSAIDs, making it difficult to avoid the ongoing use of NSAIDs.

A retrospective study published in the American Journal of Medicine in 2021, including a total of 31,340 acute pancreatitis patients, found that prior exposure to non-steroidal anti-inflammatory drugs reduces the rate of organ failure and in-hospital mortality in acute pancreatitis (Ladd et al., 2022). Therefore, treatment with NSAIDs before and during the early stages of AKI decreased the chance of developing AKI, which might be related to NSAIDs preventing organ failure. At this time, the precise mechanism by which NSAIDs exert this beneficial effect was unknown. NSAIDs were best known for inhibiting cyclooxygenases, which produce prostaglandins and thromboxanes. Its anti-inflammatory effects extend far beyond cyclooxygenase inhibition, including the inhibition of the pivotal nuclear factor kappa-light-chain-enhancer of activated B cells (NF-B) and the induction of certain types of eicosanoids, which reduced pro-inflammatory mediators such as TNF-, IL-1, and IL-6 (Deligiannidou et al., 2021; Pekacar et al., 2021; Ladd et al., 2022).

### Strength of the Study

1) AKI could be diagnosed intermittently in this study using a “pop-up” alert system based on the electronic health record, thereby avoiding missed or delayed diagnosis of AKI; 2) the data for this study were derived from prospective RCT research, and the collected indicators were all objective, ensuring the data’s authenticity and reliability; 3) prior research on NSAIDs had primarily focused on determining if they enhance the risk of AKI. However, little data exist on the optimal usage of NSAIDs in individuals with AKI. This study had practical and educational implications for the appropriate usage of NSAIDs in patients with AKI.

### Limitations to the Study

1) The study’s applicability was restricted since the original data did not include the particular medication property name, dosage, and duration of usage for NSAIDs and co-administration of other nephrotoxic medications, including beta lactams and glycopeptides. As a result, further studies were needed to determine the relationship between the different types, dosages, and duration of usage of NSAIDs, and co-administration of other nephrotoxic medications and the outcome of individuals with AKI. 2) The follow-up period was relatively short. It might result in an underestimate of the risk of mortality associated with NSAIDs in patients with AKI. It was required to perform a longer follow-up to elucidate the NSAID mortality risk better. 3) In addition, the reason for admission was not provided, which might also present a potential risk of bias.

## Conclusion

Before the onset of AKI, NSAID treatment might be safe, but during the early onset of AKI, it might hasten the progression of AKI and worsen the patient’s prognosis, especially in patients with age ≥ 65 years, CKD, congestive heart failure, hypertension, and liver disease. For the rational use of NSAIDs, the vigilance of AKI should be strengthened; the timely detection of AKI patients and the critical monitoring of special populations were important means to avoid NSAID-related side effects.

## Data Availability

The datasets presented in this study can be found in online repositories. The names of the repository/repositories and accession number(s) can be found below: https://datadryad.org/stash/dataset/doi:10.5061/dryad.4f4qrfj9.

## Abbreviations

NSAIDs, non-steroidal anti-inflammatory drugs; MAP, mean arterial pressure; CKD, chronic kidney disease; COPD, chronic obstructive pulmonary disease; BUN, blood urea nitrogen; SOFA, sequential organ failure assessment; AKI, acute kidney injury; PPI, proton pump inhibitor; ACEi, angiotensin-converting enzyme inhibitor; ARB, angiotensin receptor blocker; KDIGO, Kidney Disease: Improving Global Outcomes; ICUs, intensive care units.
